# Astrocyte secretome remodeling under iron deficiency: potential implications for brain iron homeostasis

**DOI:** 10.1242/bio.062057

**Published:** 2025-07-09

**Authors:** Mariam Duhaini, Habiba S. Shamroukh, Zhi Zhang, Kalyan C. Kondapalli

**Affiliations:** Department of Natural Sciences, University of Michigan-Dearborn, 4901 Evergreen Road, Dearborn, MI 48128, USA

**Keywords:** Astrocyte, Secretome, Iron deficiency, Brain iron homeostasis, Proteomics, Metabolic reprogramming

## Abstract

The brain is the most metabolically active organ in the body and has a high demand for iron. Iron deficiency impairs brain function and is linked to various neurological disorders. To maintain iron homeostasis, astrocytes respond to iron levels and signal brain microvascular endothelial cells (BMVECs), which regulate iron import into the brain. However, the specific signaling molecules released by astrocytes remain largely unknown. In this study, we addressed this by performing a global proteomic analysis of the secretome of primary mouse astrocytes cultured under iron-deficient conditions. Quantitative mass spectrometry demonstrated significant remodeling of the astrocyte secretome in response to iron deficiency, affecting critical pathways related to metabolic reprogramming, stress responses, and cellular communication. We identified specific secreted factors with potential roles in paracrine signaling, with their secretion supported by prediction analysis. Our analysis also revealed novel condition-specific proteins. These findings provide new insights into astrocyte communication under iron stress and its potential influence on iron availability at the blood–brain barrier. This study establishes a foundation for future investigations into astrocyte-secreted factors and their roles in neurological diseases associated with iron dysregulation.

## Teaser

**Summary:** This study identifies new molecular signals released by brain astrocytes under iron deficiency. These findings offer crucial insights into brain iron control and its relevance to neurological health.

## INTRODUCTION

Iron is essential for normal brain function (Beard and Connor, 2003). It is central to oxidative metabolism and is a cofactor in the synthesis of neurotransmitters and myelin (Beard and Connor, 2003). Maintaining precise iron homeostasis within the central nervous system (CNS) is crucial, as both iron deficiency and overload can lead to a range of neurological dysfunctions (Rouault, 2013). The blood-brain barrier (BBB), a dynamic interface composed of brain microvascular endothelial cells (BMVECs) and supporting glial cells, particularly astrocytes, plays a critical role in regulating iron entry into the brain parenchyma (McCarthy and Kosman, 2015; Duck and Connor, 2016).

Astrocytes are the most abundant glial cells in the brain and contain iron concentrations approximately twice that of neurons (Reinert et al., 2019). They play a central role in BBB function and extensively communicate with brain microvascular BMVECs through the secretion of various factors (Beydoun et al., 2017; McCarthy and Kosman, 2014, 2012; Simpson et al., 2015). This astrocyte–BBB signaling is essential for regulating iron transport proteins and maintaining brain iron homeostasis (Beydoun et al., 2017; McCarthy and Kosman, 2014, 2012; Simpson et al., 2015). Despite the critical roles of astrocytes in iron regulation, the impact of iron deficiency on their secretome remains largely unexplored. A detailed characterization of the astrocyte secretome will provide a foundation for uncovering novel signaling pathways and therapeutic targets for neurological disorders associated with iron dysregulation, including iron deficiency anemia, restless legs syndrome, Parkinson's disease, and Alzheimer's disease (Rouault, 2013). Importantly, while differential expression analysis reveals changes in shared proteins, focusing solely on common proteins may overlook key secreted factors that are completely absent in one condition but detectable in another. Such ‘on/off’ changes in secretory output can represent some of the most significant biological shifts and act as highly important drivers of the cellular response.

To address this gap, we conducted what is, to our knowledge, the first global proteomic analysis of the secretome of primary mouse astrocytes under iron-deficient conditions. It is important to note that our study models iron deficiency, an environment of reduced iron availability, rather than complete iron depletion, which could be detrimental to cell viability (Simpson et al., 2015; Bajbouj et al., 2018). Our study revealed substantial changes in secreted proteins involved in cellular metabolism, iron homeostasis, and cell signaling. We also examined predicted secretion pathways, highlighting a potential role for non-classical mechanisms in the release of specific functional protein categories. These findings provide new insights into the molecular response of astrocytes to iron deficiency, suggesting active remodeling of their extracellular environment to influence iron availability and signaling at the BBB.

## RESULTS

### Iron deficiency remodels the astrocyte secretome: key protein changes

**Fig. 1.**
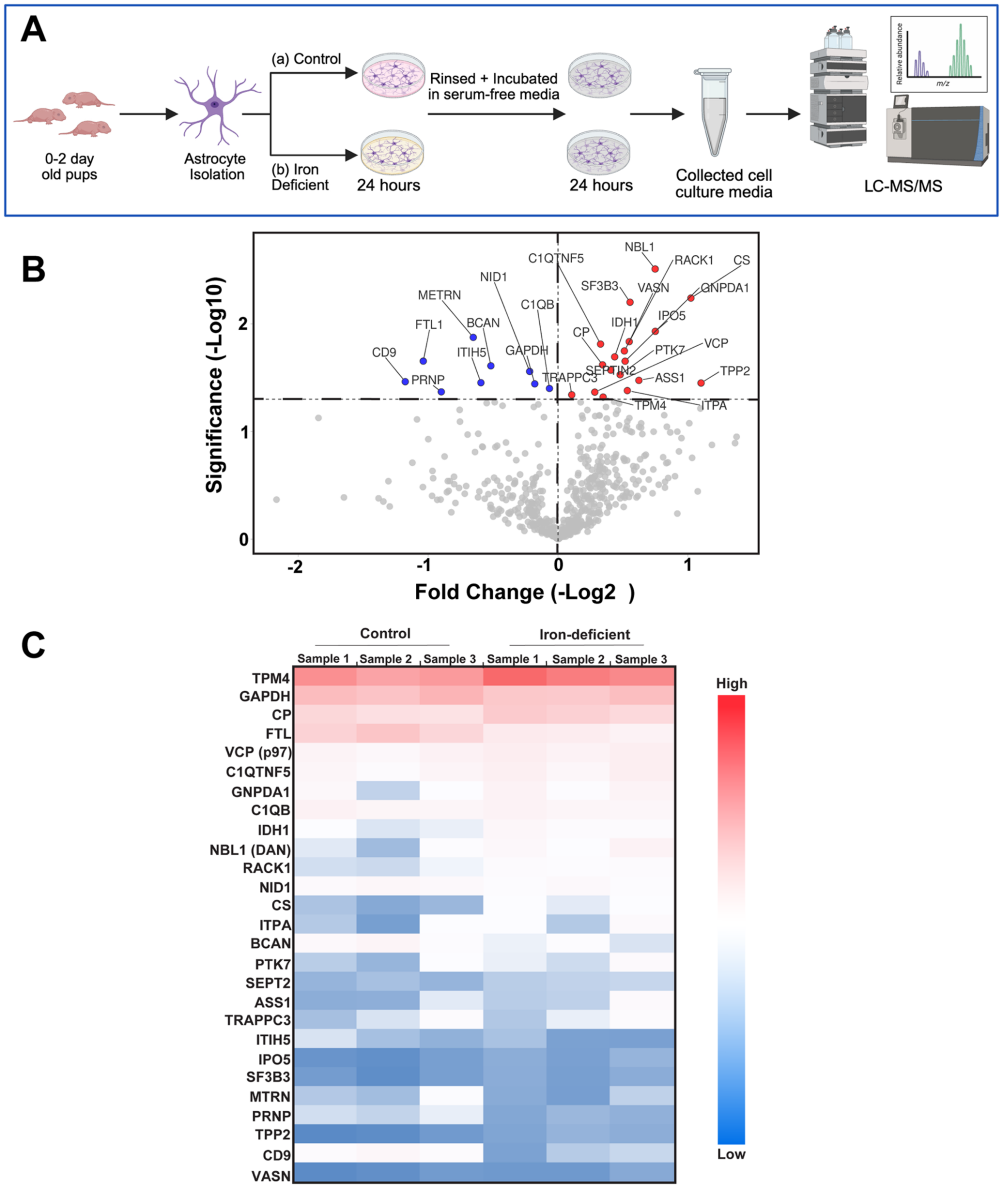
**Experimental workflow and differential analysis of the astrocyte secretome under iron-deficient conditions.** (A) Schematic of the experimental design. Primary astrocytes were isolated from neonatal mouse pups and cultured under control or iron-deficient conditions for 24 h, followed by collection of conditioned media and analysis by liquid chromatography-tandem mass spectrometry (LC-MS/MS). (B) Volcano plot showing differential protein secretion between iron-deficient and control astrocytes. The x-axis represents log_2_ fold change, and the y-axis shows −log_10_(*P*-value). Red points indicate proteins significantly upregulated, blue points indicate proteins significantly downregulated (*P*<0.05 threshold), and gray points indicate non-significant proteins. (C) Heatmap displaying the relative abundance of proteins with significantly altered secretion. Rows represent individual proteins and columns represent biological replicates. The color intensity represents relative expression: blue indicates lower expression, white/light tones indicate intermediate expression, and red indicates higher expression, all relative to the mean normalized spectral abundance factor (NSAF) value of each protein across all samples. Proteins are sorted from top to bottom based on their average relative expression in the iron-deficient condition, from highest to lowest.

Primary mouse astrocytes were cultured for 24 h under iron-deficient or control conditions. Conditioned media were processed by SDS-PAGE (Fig. S1) and analyzed by nano-liquid chromatography-tandem mass spectrometry (LC-MS/MS) (Fig. 1A). Comparative proteomics revealed 921±232 proteins in control cultures and 983±110 proteins in iron-deficient cultures (mean±s.d., *n*=3 biological replicates). A subset of 542 proteins common to all samples was used for quantitative analysis (Table S1). Volcano plot analysis (Fig. 1B) identified 27 proteins significantly differentially secreted (*P*<0.05), with 18 upregulated and nine downregulated under iron deficiency. The full list of these 27 differentially expressed proteins, along with their fold changes and *P*-values, is presented in Table 1. The differential expression of these 27 proteins is visually represented in the heatmap (Fig. 1C). This heatmap displays the relative protein expression levels across all six samples (three control and three iron-deficient), allowing for a comparison of expression patterns between the two conditions. Further analysis identified four key proteins with substantial changes, representing diverse functional categories relevant to the astrocyte response to iron deficiency: tripeptidyl-peptidase 2 (Tpp2; 2.14-fold increase), citrate synthase (Cs; 2.03-fold increase), CD9 antigen (Cd9; 0.44-fold decrease), and ferritin light chain 1 (Ftl1; 0.48-fold decrease). These findings demonstrate that iron deficiency induces significant and complex remodeling of the astrocyte secretome, impacting proteins involved in metabolism, iron regulation, and intercellular communication.

**Table 1.** Differentially secreted proteins in astrocyte secretome under iron deficiency

Name	Accession number and symbol	Fold change	Significance (*P*-value)
Tripeptidyl-peptidase 2	|Q64514|TPP2	2.15	0.035
Citrate synthase, mitochondrial	|Q9CZU6|CIS	2.03	0.006
Importin-5	|Q8BKC5|IPO5	1.68	0.012
Neuroblastoma suppressor of tumorigenicity	|Q61477|NBL1	1.68	0.003
Arginosuccinate synthase	|P16460|ASS1	1.54	0.033
Splicing factor 3B subunit 3	|Q921M3|SF3B3	1.47	0.006
Vasorin	|Q9CZT5|VASN	1.46	0.015
Inosine triphosphate pyrophosphatase	|Q9D892|ITPA	1.45	0.042
Glucosamine-6-phosphate isomerase 1	|O88958|GNPI1	1.43	0.022
Receptor of activated protein C kinase 1	|P68040|RACK1	1.43	0.018
Inactive tyrosine-protein kinase 7	|Q8BKG3|PTK7	1.39	0.030
Isocitrate dehydrogenase [NADP] cytoplasmic	|O88844|IDH1	1.35	0.020
Septin-2	|P42208|SEPT2	1.33	0.027
Tropomyosin alpha-4 chain	|Q6IRU2|TPM4	1.27	0.048
Ceruloplasmin	|Q61147|CP	1.27	0.024
Complement C1q tumor necrosis factor-related protein 5	|Q8K479|C1QTNF5	1.25	0.015
Transitional endoplasmic reticulum ATPase	|Q01853|VCP	1.22	0.043
Trafficking protein particle complex subunit 3	|O55013|TRAPPC3	1.08	0.046
Complement C1q subcomponent subunit B	|P14106|C1QB	0.96	0.040
Glyceraldehyde-3-phosphate dehydrogenase	|P16858|GAPDH	0.88	0.036
Nidogen-1	|P10493|NID1	0.86	0.028
Brevican core protein	|Q61361|PGCB	0.70	0.025
Inter-alpha-trypsin inhibitor heavy chain H5	|Q8BJD1|ITIH5	0.66	0.035
Meteorin	|Q8C1Q4|METRN	0.64	0.013
Major prion protein	|P04925|PRNP	0.54	0.043
Ferritin light chain 1	|P29391|FTL1	0.49	0.022
CD9 antigen	|P40240|CD9	0.44	0.034

Fold change represents the fold change in protein abundance in iron-deficient conditions relative to control. Values greater than 1 indicate upregulation, while values less than 1 indicate downregulation. Statistical significance was defined as *P*<0.05. Protein abundance was quantified using NSAF values.

### A subset of differentially secreted astrocyte proteins utilize non-classical pathways

To gain insight into the mechanisms underlying the presence of these differentially expressed proteins in the astrocyte secretome, we examined their predicted secretion routes using SecretomeP (Table S2). Among these 27 proteins, nine, including factors involved in pathways highlighted in subsequent analysis, met the criteria for non-classical secretion (Table S2). For example, Ceruloplasmin, a key protein in iron export, is predicted to be secreted non-classically. Similarly, nidogen-1 and inter-alpha trypsin inhibitor, proteins potentially involved in extracellular matrix organization, also show high neural network (NN) scores for non-classical secretion. The presence of complement C1q-related proteins, which could influence immune responses, further suggests that non-classical secretion is a feature of several differentially secreted proteins involved in key functional categories.

These findings indicate that a significant subset of the astrocyte proteins that are differentially secreted under iron deficiency are predicted to utilize non-classical secretory pathways, suggesting that these unconventional routes play a substantial role in shaping the astrocyte secretome during iron stress.

### Iron deficiency induces broad metabolic reprogramming and functional alterations in the astrocyte secretome

**Fig. 2.**
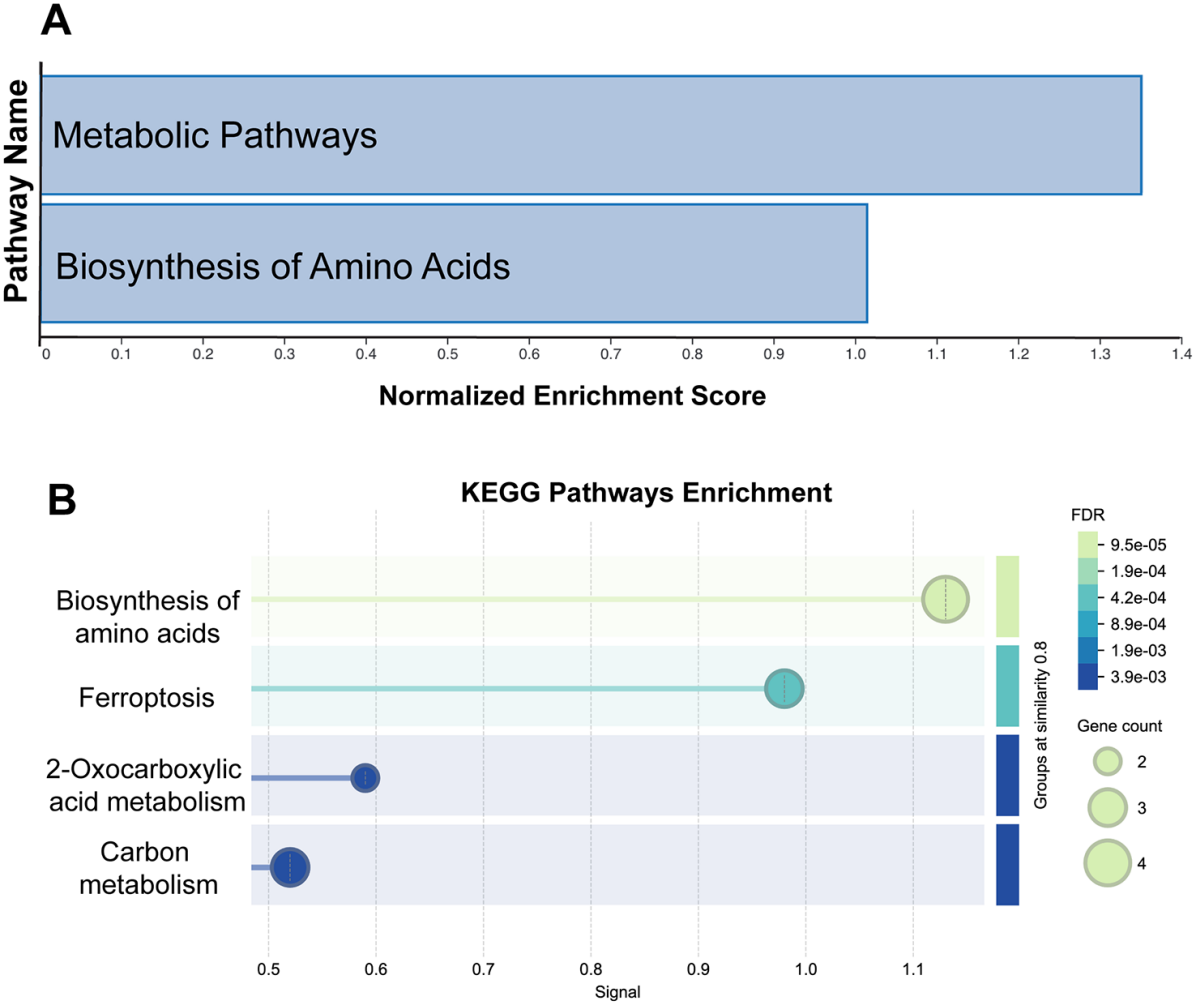
**KEGG pathway enrichment analysis highlights core metabolic pathways associated with astrocyte secretome proteins under iron deficiency.** (A) Bar graph depicting the normalized enrichment scores for key pathways associated with the astrocyte secretome under iron deficiency conditions. The metabolic pathways and biosynthesis of amino acids are highlighted, indicating significant enrichment. (B) Bubble plot representing the KEGG pathways enrichment in the astrocyte secretome. Pathways are plotted on the y-axis, with corresponding signals on the x-axis. The size of each bubble corresponds to the gene count within the pathway, while the color gradient indicates the false discovery rate (FDR), with darker shades representing more significant enrichment.

**Fig. 3.**
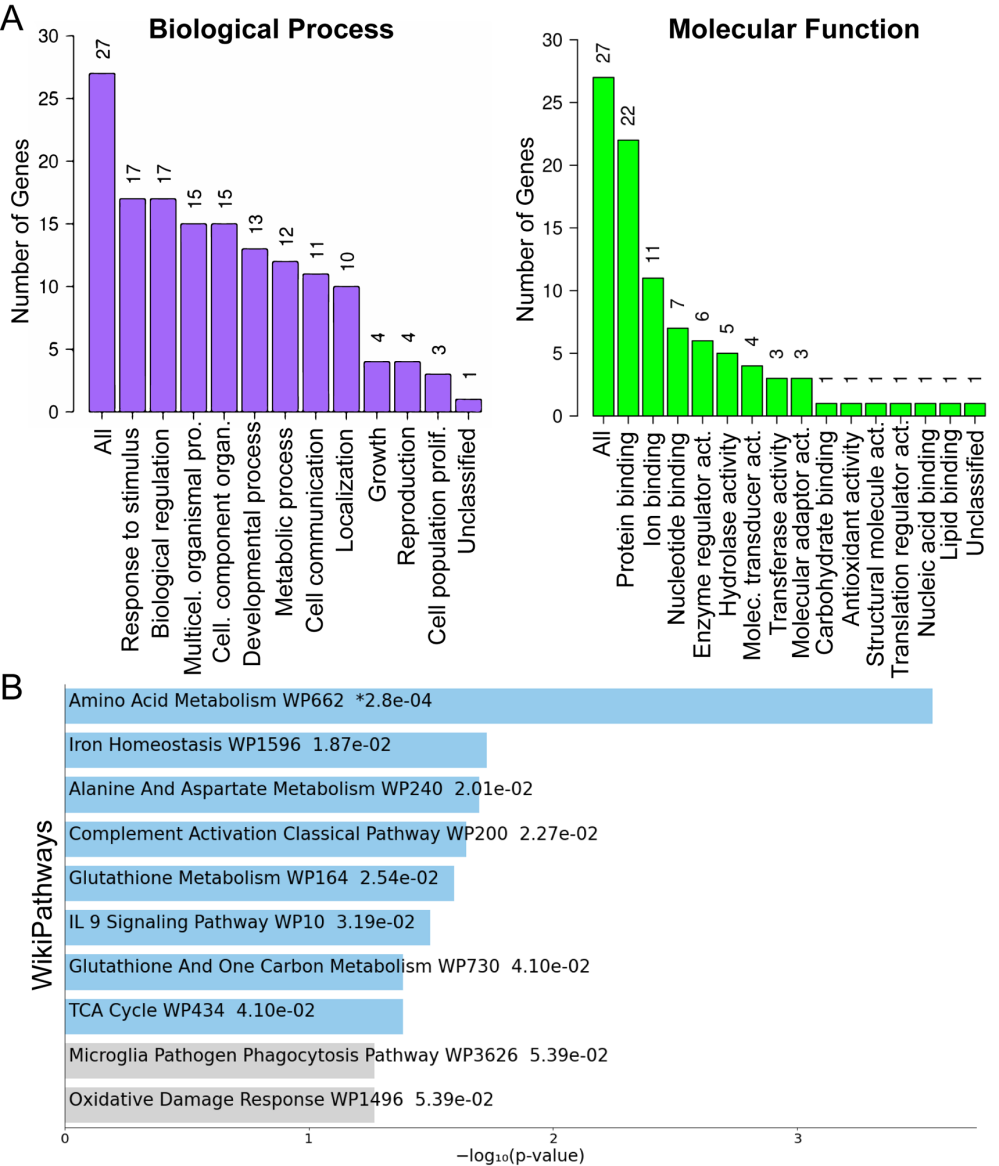
**Gene Ontology and WikiPathways enrichment analyses highlight functional pathways associated with astrocyte secretome proteins under iron deficiency.** (A) Gene Ontology (GO) biological process enrichment analysis showing significantly enriched categories among differentially expressed proteins in the astrocyte secretome. The numbers on top of each bar indicate the number of genes associated with each category, with key processes such as response to stimulus and biological regulation. (B) GO molecular function enrichment analysis depicting essential functional categories. The numbers above each bar represent the number of genes involved in categories such as protein binding and catalytic activity. (C) WikiPathways analysis identifying enriched pathways, with numbers adjacent to each bar indicating the −log10(*P*-value) of significance for pathway enrichment.

To gain insight into the functional consequences of astrocyte secretome changes under iron deficiency, we performed Kyoto Encyclopedia of Genes and Genomes (KEGG) and Gene Ontology (GO) pathway enrichment analyses. KEGG analysis identified significant enrichment of proteins associated with several metabolically relevant pathways, including biosynthesis of amino acids, ferroptosis, 2-oxocarboxylic acid metabolism, and carbon metabolism (Fig. 2A,B). GO analysis further supported these findings, highlighting enriched biological process categories such as, response to stimulus, response to organic substance, metabolic process, and cell communication (Fig. 3A). Similarly, enriched molecular function categories included, binding, catalytic activity, and transporter activity (Fig. 3B). Notably, WikiPathways analysis also showed enrichment for amino acid metabolism and iron homeostasis pathways (Fig. 3C). Collectively, these enrichment analyses based on the secretome indicate that iron deficiency may broadly impact astrocyte metabolism and iron handling, suggesting comprehensive metabolic reprogramming and altered functional roles beyond classical iron handling in response to iron deprivation.

### Network analysis reveals coordinated functional modules and predicted transcriptional regulation of the astrocyte secretome

**Fig. 4.**
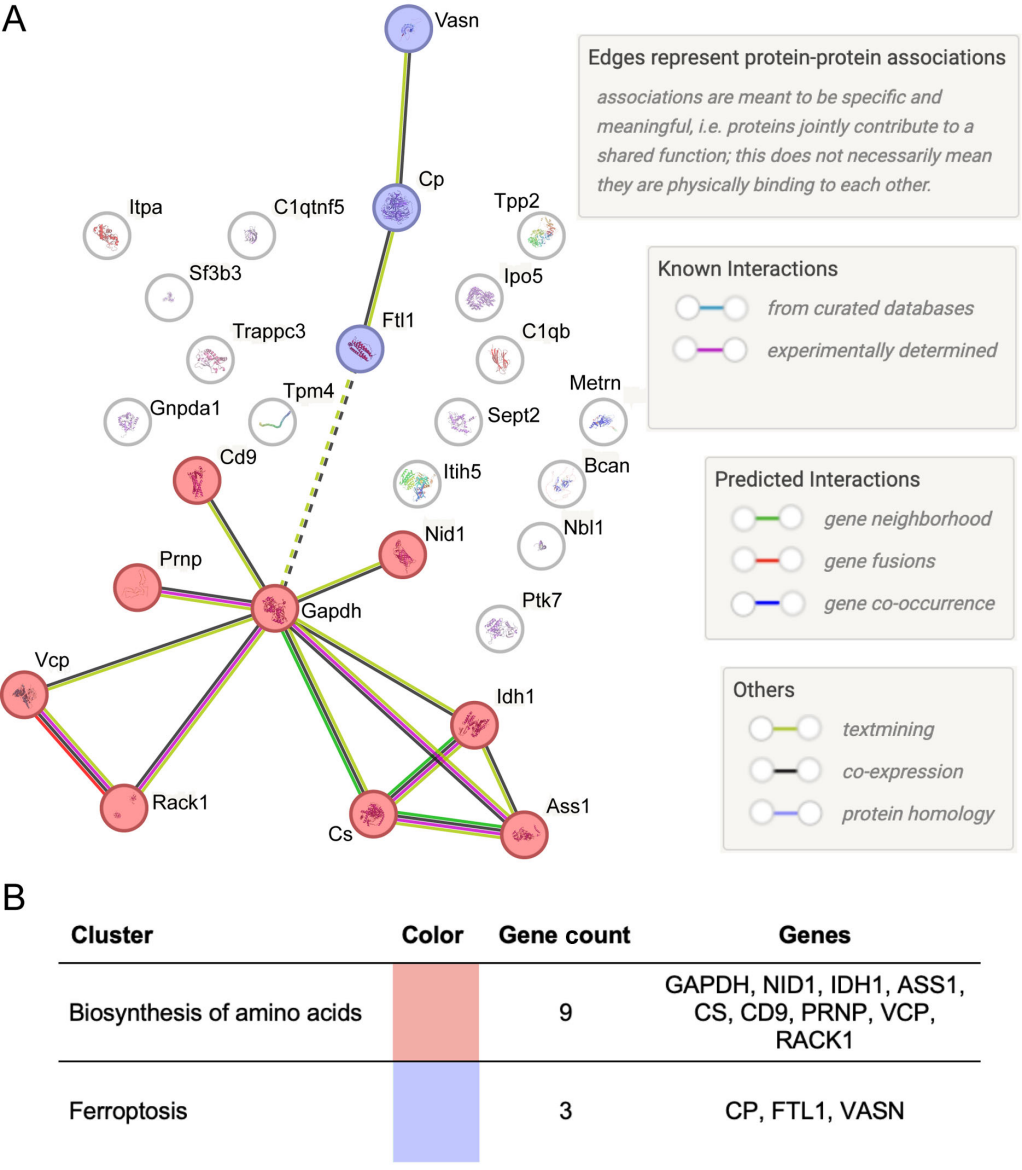
**Protein–protein interaction network and functional clustering associated with astrocyte secretome under iron deficiency.** (A) Protein–protein interaction network of differentially secreted astrocyte proteins visualized using STRING. The analysis identified functional clusters using MCL, highlighting distinct biological functions. Nodes represent proteins, and edges (lines connecting the nodes) depict their associations. The analysis revealed two prominent clusters: Cluster 1 in pink and Cluster 2 in purple. (B) Summary table of identified clusters. Cluster 1 (biosynthesis of amino acids) comprises nine proteins potentially involved in metabolic adaptations, while Cluster 2 (ferroptosis) includes three proteins implicated in ferroptotic signaling pathways. These clusters illustrate coordinated regulatory responses in astrocytes when faced with iron deficiency.

To further elucidate the coordinated biological functions underlying the astrocyte secretome response to iron deficiency, we performed Markov Clustering (MCL) on the protein–protein interaction (PPI) network of the 27 differentially secreted proteins using STRING. This analysis revealed two prominent clusters representing functionally enriched modules (Fig. 4). Cluster 1 consisted of nine proteins, including GAPDH, RACK1, PRNP, CS, IDH1, and ASS1, and was associated with the biosynthesis of amino acids. This suggests that astrocytes undergo metabolic reprogramming in response to iron deprivation, possibly to support the energy demands of stress adaptation and signaling. Cluster 2 contained three proteins: ceruloplasmin (Cp), FTL1, and VASN, which are associated with ferroptosis. The inclusion of these iron-responsive proteins in a discrete cluster implies that iron deficiency triggers the secretion of molecules involved in iron scavenging, oxidative stress regulation, and ferroptotic signaling. These modules illustrate coordinated regulation and provide mechanistic insight into astrocyte responses to iron deficiency.

**Fig. 5.**
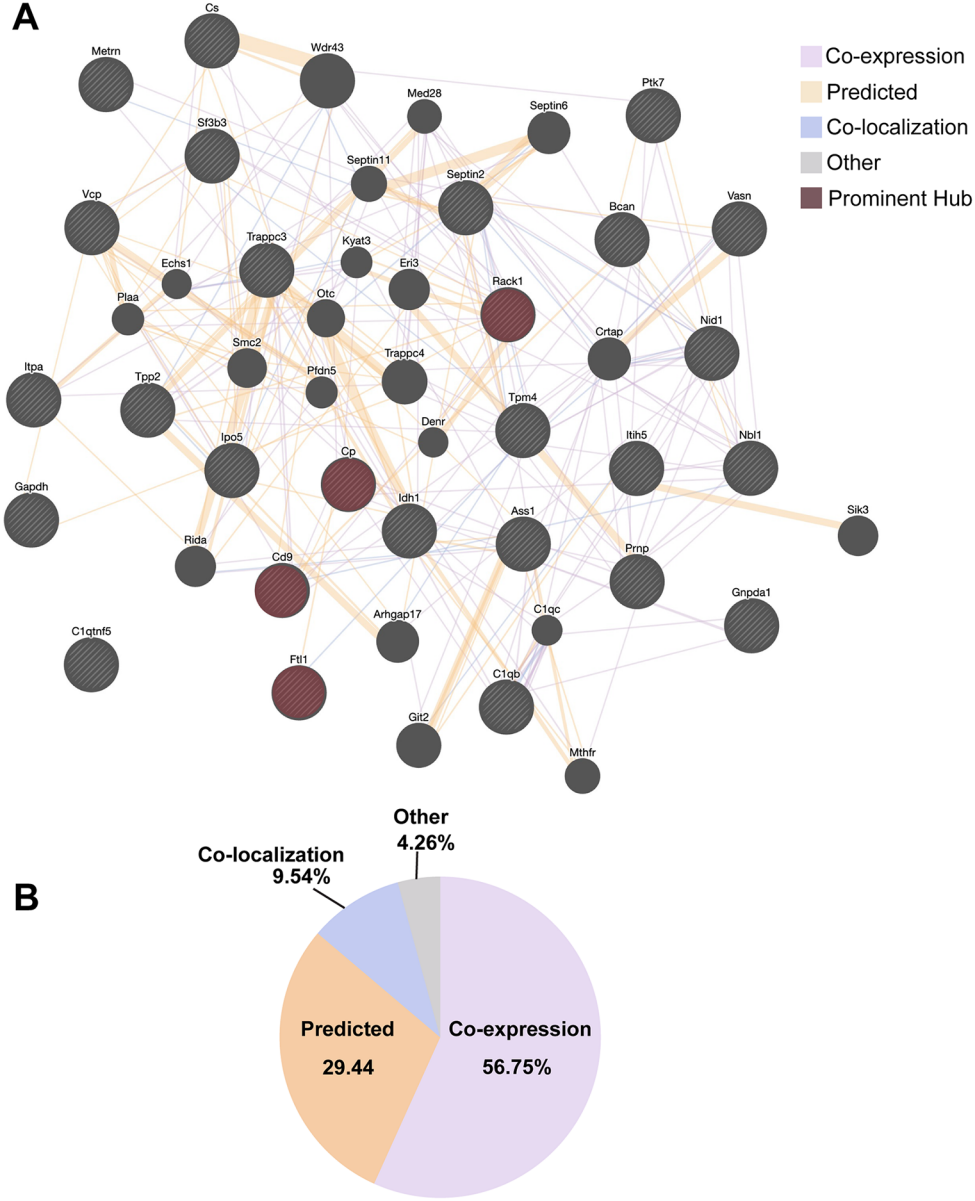
**Network analysis and interaction type contribution associated with astrocyte secretome under iron deficiency.** (A) GeneMANIA network visualization displaying protein-protein interactions among differentially secreted astrocyte proteins under iron deficiency. Nodes represent proteins, with striped or hatched circles indicating query nodes (input proteins) and solid circles representing GeneMANIA-added intermediate nodes. Edges (lines connecting nodes) depict various interaction types, color-coded for clarity: co-expression (purple), predicted interactions (orange), co-localization (blue), and other types (gray). Prominent hub proteins, such as Cp, Ftl1, Cd9, and Rack1, are highlighted in brown, indicating their central role in the interaction network. (B) Pie chart illustrating the proportional contribution of different interaction types to the overall network. Co-expression interactions make up the largest share (56.75%), followed by predicted (29.44%), co-localization (9.54%), and other interaction types (4.26%).

In order to understand the broader interaction landscape among the secreted proteins, we conducted a GeneMANIA network analysis using the 27 differentially secreted astrocyte proteins as input. The resulting network revealed extensive functional connectivity, with 56.75% of interactions attributed to co-expression, suggesting shared biological processes or regulatory mechanisms (Fig. 5A,B). An additional 29.44% of interactions were attributed to predicted functional associations. Prominent hubs within the network included Cp, Ftl1, CD9, and RACK1, suggesting their central roles in orchestrating the astrocyte response to iron deficiency (Fig. 5A,B). Co-localization and shared pathway data supported interactions between secretory factors involved in extracellular matrix organization (Nid1, Itih5), redox regulation (Vcp, Idh1), and vesicle trafficking (Trappc3, Cd9), indicating that these proteins are not regulated in isolation but rather as part of a coordinated, stress-responsive signaling network (Fig. 5A,B). These findings further support the existence of co-regulated secretory programs during iron deficiency.

**Fig. 6.**
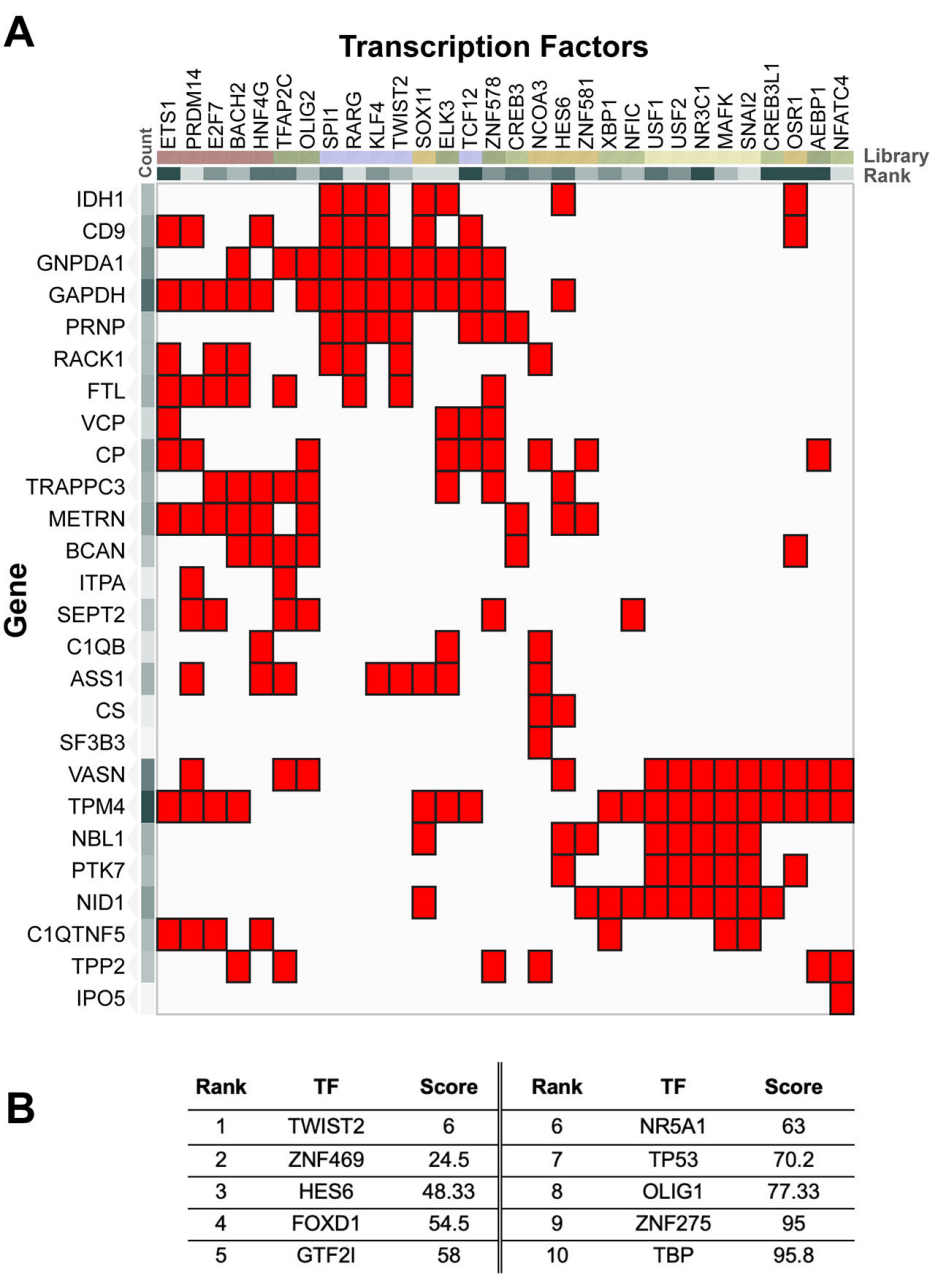
**Enrichment and network analysis of transcription factors associated with astrocyte secretome proteins under iron deficiency.** (A) Heatmap presenting the enrichment of transcription factor binding sites across differentially secreted astrocyte proteins. Rows correspond to genes, and columns represent transcription factors (TFs) from various libraries. Each library is indicated by the following identifiers: ARCHS4 (e.g. NFATC4, AEBP1, OSR1), ENCODE (e.g. MAFK, NR3C1, USF2), Enrichr (e.g. XBP1, ZNF581, HES6), GTEx (e.g. ZNF578, TCF12, ELK3), Literature (e.g. KLF4, RARG, SPI1), and ReMap (e.g. HNF4G, BACH2, E2F7). Red squares indicate significant enrichment of TF binding sites, suggesting potential regulatory interactions influenced by iron deficiency. (B) Table listing the top ten ranked transcription factors based on enrichment scores. Each entry includes the rank, TF name, and score, with TWIST2 and ZNF469 identified as the most enriched TFs. This highlights potential key regulators of the astrocyte response to iron deficiency.

To identify potential upstream regulators of this secretory response, we next performed transcription factor enrichment analysis using the ChEA3 platform. The analysis, integrating multiple libraries including GTEx co-expression, ENCODE ChIP-seq, and literature-curated sources, identified over 1600 predicted transcription factors regulating the secretome gene set. The top-ranking transcription factors predicted to regulate the secretome gene set are presented in Fig. 6, and include TWIST2, ZNF469, HES6, FOXD1, GTF2I, NR5A1, and TP53. The complete list of predicted transcription factors, along with their scores and associated libraries, is provided in Table S3. Notably, several of these transcription factors are implicated in oxidative stress responses (TP53, HES6), astrocyte differentiation (FOXD1, TWIST2), and metabolic regulation (GTF2I, NR5A1). Their predicted involvement suggests that the observed secretome remodeling is under coordinated transcriptional control, integrating metabolic and stress-related signaling cascades.

### Identification of condition-exclusive secretome proteins in astrocyte cultures

To further address the comprehensive composition of the astrocyte secretome under varying iron levels, we analyzed proteins exclusively detected in either control or iron-deficient culture conditions. Our analysis identified 181 proteins uniquely present in the secretome of control astrocyte cultures. Of these, neurofascin (Nfasc), an extracellular adhesion molecule critical for nervous system development and function, was the only protein found to be statistically significant. Similarly, we identified 158 proteins exclusively detected in the secretome of iron-deficient astrocyte cultures. Among these, 11 proteins were found to be statistically significant. These 12 statistically significant proteins (Nfasc from control, and the 11 from iron-deficient cultures), which include diverse functional categories such as metabolic enzymes, cytoskeletal components, and signaling molecules, are summarized in Table 2. The complete list of all proteins exclusively detected in either control (181 proteins) or iron-deficient (158 proteins) secretomes, along with their detection status across replicates, is provided in Table S4.

**Table 2.** Condition-exclusive secretome proteins in astrocyte cultures

Name	Accession number and symbol	Unique to condition	Significance (*P*-value)
Beta-actin-like protein 2	|Q8BFZ3|ACTBL	Iron deficient	0.0002
3-hydroxyisobutyrate dehydrogenase, mitochondrial	|Q99L13|3HIDH	Iron deficient	0.0013
Ribonucleoside-diphosphate reductase subunit M2	|P11157|RIR2	Iron deficient	0.0015
DNA-directed RNA polymerases I, II, and III subunit RPABC3	|Q923G2|RPAB3	Iron deficient	0.0022
ATP-dependent 6-phosphofructokinase, liver type	|P12382|PFKAL	Iron deficient	0.0028
Talin-2	|Q71LX4|TLN2	Iron deficient	0.0055
Ribosome maturation protein SBDS	|P70122|SBDS	Iron deficient	0.0065
Eukaryotic translation initiation factor 3 subunit M	|Q99JX4|EIF3M	Iron deficient	0.0075
Semaphorin-7A	|Q9QUR8|SEM7A	Iron deficient	0.0214
Phosphoribosylformylglycinamidine synthase	|Q5SUR0|PUR4	Iron deficient	0.0334
Vascular endothelial growth factor A	|Q00731|VEGFA	Iron deficient	0.0488
Neurofascin	|Q810U3|NFASC	Control	0.0062

Proteins were identified as statistically significant if their *P*<0.05.

## DISCUSSION

The brain requires a precise balance of iron for proper functioning, as both deficiency and excess can lead to neurological dysfunction (Rouault, 2013). The BBB, consisting of BMVECs and astrocytes, serves as the primary interface controlling iron entry into the brain (McCarthy and Kosman, 2015). Astrocytes are critical cellular components of this interface, communicating with BMVECs via secreted factors to regulate the expression and activity of iron transport proteins (McCarthy and Kosman, 2015). Despite the recognized importance of astrocyte-BBB signaling in iron homeostasis, a comprehensive understanding of how the astrocyte secretome is remodeled under low iron conditions is missing. Therefore, we conducted a proteomic analysis of the astrocyte secretome to provide the first global analysis of the astrocyte response to iron deficiency, revealing substantial changes in proteins associated with metabolic processes, iron homeostasis, and oxidative stress regulation.

Our proteomic analysis revealed significant remodeling of the astrocyte secretome under low iron conditions, affecting proteins involved in energy metabolism, iron handling, and cell signaling. Notably, increases in citrate synthase (2.03-fold) and tri-peptidyl peptidase 2 (TPP2; 2.15-fold) suggest metabolic adaptation, while changes in Cp (1.27-fold increase) and Ftl1 (0.49-fold decrease) point to altered iron management. Alterations in signaling molecules such as CD9 antigen (0.44-fold decrease) and RACK1 (1.08-fold increase) further suggest potential impacts on communication with the BBB.

Beyond the quantitative changes in proteins common to both conditions, our analysis of condition-exclusive secretome components provides further critical insights into the distinct adaptive responses of astrocytes to varying iron levels. This approach was essential, as focusing solely on differentially abundant proteins might overlook key proteins that are exclusively detected or completely absent in a given condition, representing some of the most significant changes in the secretory output and cellular biology. Such a comprehensive analysis is crucial for identifying these ‘on/off’ switches in the secretome, as they represent significant drivers of the cellular response to iron status and can unveil entirely new pathways or mechanisms involved in the astrocyte response to iron deficiency.

In control astrocyte secretomes, Nfasc, an extracellular adhesion molecule vital for nervous system development and glia-neuron interactions, was uniquely and significantly detected, suggesting its specific role in maintaining the normal basal secretome profile. Conversely, the iron-deficient secretome exhibited 11 statistically significant unique proteins, encompassing diverse functional categories such as metabolic enzymes, cytoskeletal components, and crucial signaling molecules. Notably, the exclusive presence of vascular endothelial growth factor A (VEGFA) in iron-deficient secretomes is particularly compelling. VEGFA is a potent angiogenic factor, but also plays significant roles in neuroprotection, blood-brain barrier integrity, and glial cell function, suggesting its induction as a specific adaptive response to iron scarcity in the brain. Similarly, the appearance of metabolic enzymes like ATP-dependent 6-phosphofructokinase, liver type (Pfkl), points towards specific metabolic rewiring in astrocytes under iron-deficient conditions. These findings underscore the dynamic and multifaceted nature of the astrocyte secretome, revealing unique protein contributions that provide a more complete picture of their adaptive strategies and potential compensatory mechanisms in response to altered iron homeostasis.

The presence of several differentially expressed proteins predicted to be secreted via non-classical mechanisms, such as Cp, nidogen-1, inter-alpha trypsin inhibitor, and complement C1q-related proteins, suggests a broader range of secretory strategies employed by astrocytes. This implies that astrocytes may utilize extracellular vesicles, ectodomain shedding, or direct plasma membrane translocation to release key signaling and structural proteins under iron-deficient conditions. The involvement of non-classical secretion pathways highlights the complexity of astrocyte responses and suggests regulatory mechanisms extending beyond conventional ER-Golgi trafficking.

The observed increase in astrocyte Cp secretion under low iron conditions warrants further attention. Cp, a key ferroxidase, facilitates iron efflux from BMVECs by oxidizing exported Fe^2+^ to Fe^3+^ (McCarthy and Kosman, 2014). Iron is exported from BMVECs via ferroportin on their basolateral surface, and Cp-mediated oxidation enables Fe^3+^ binding to transferrin in the interstitial fluid, supporting neuronal and glial iron uptake through transferrin receptors (McCarthy and Kosman, 2015). Increased Cp secretion into the astrocyte microenvironment could thus enhance iron availability to brain cells during deficiency. This mechanism aligns with previous reports on astrocyte regulation of iron homeostasis. Although the fold change in Cp secretion was modest, it may still have physiological significance given the localized nature of signaling within the neurovascular unit.

Conversely, we observed a significant downregulation of FTL1 in the astrocyte secretome under iron-deficient conditions, indicating reduced extracellular release of this protein. Although seemingly counterintuitive, this decrease likely reflects a cellular strategy to conserve iron for intracellular metabolic needs. Under low iron availability, increased activity of iron regulatory proteins inhibits ferritin mRNA translation, reducing both intracellular ferritin synthesis and secretion (McCarthy and Kosman, 2015). It is also possible that astrocytes selectively secrete FTL1 under iron-deficient conditions to redistribute limited iron to other brain cells or for signaling purposes, suggesting distinct regulation of secreted versus intracellular ferritin pools. The coordinated downregulation of ferritin secretion alongside the upregulation of Cp, a facilitator of iron export, implies that astrocytes prioritize immediate iron release over extracellular iron storage under deficiency. Notably, astrocyte ferritin secretion has been shown to increase under iron overload, supporting the dynamic regulation of ferritin trafficking in response to iron availability.

In addition to changes in iron handling proteins, we also observed significant alterations in proteins related to energy metabolism, suggesting that astrocytes undergo metabolic adaptations in response to iron deficiency. The significant upregulation of Cs, a key enzyme in the mitochondrial TCA cycle, suggests that astrocytes increase their metabolic activity to compensate for the potential reduction in ATP production due to iron deficiency, as iron is a crucial cofactor for several mitochondrial enzymes. This observation aligns with the findings of Pino et al., who reported altered Cs activity in the striatum of iron-deficient rats, further supporting the link between iron deficiency and altered brain energy metabolism (Pino et al., 2022). The presence of Cs in the secretome is intriguing, as it is traditionally considered a mitochondrial enzyme. However, evidence suggests that Cs can have ‘moonlighting’ functions and has been identified in extracellular fluids and the extracellular space according to databases such as the Human Protein Atlas and UniProtKB (Buschow et al., 2010; UniProt Consortium, 2025). While its extracellular role is not well-characterized, it may represent a previously unknown function triggered by iron-deficient conditions. It is important to note that some of these metabolic changes may also reflect cellular adaptations to the serum-free culture conditions, particularly potential amino acid deprivation. This increased metabolic activity could be necessary to support the cellular machinery involved in sensing iron levels, synthesizing and secreting signaling molecules (like Cp), and maintaining cellular homeostasis in a low-iron environment. Furthermore, the energy demands of actively signaling to the BBB to modulate iron transport might also necessitate this metabolic upregulation.

To further elucidate the coordinated biological functions and regulatory mechanisms underlying the astrocyte secretome response to iron deficiency, we performed detailed network and upstream regulator analyses. Our MCL clustering of the protein-protein interaction network revealed distinct functional modules within the secretome. For instance, a cluster associated with biosynthesis of amino acids (including GAPDH, RACK1, PRNP, CS, IDH1, ASS1) suggests a metabolic reprogramming strategy to support stress adaptation. Concurrently, a ferroptosis cluster (comprising of Cp, FTL1, and VASN) highlights that iron deficiency triggers the secretion of molecules involved in iron scavenging and oxidative stress regulation. These findings provide compelling evidence for coordinated regulation and mechanistic insight into astrocyte responses to iron deficiency.

The GeneMANIA network analysis further elucidated the extensive functional connectivity among the secreted proteins, with a high degree of co-expression and predicted functional associations. The identification of central hub proteins such as Cp, Ftl1, CD9, and RACK1 underscores their crucial roles in orchestrating the astrocyte response. Interactions related to extracellular matrix organization, redox regulation, and vesicle trafficking supported a coordinated, stress-responsive signaling network. These results strongly demonstrate that secreted factors are not regulated in isolation but participate in highly interconnected programs to adapt to iron deficiency, thus providing a comprehensive view of the complex coordination requested by the reviewer.

Finally, our ChEA3 transcription factor enrichment analysis offered critical insights into the upstream regulatory control of the secretome remodeling. The prediction of top-ranking transcription factors, including TWIST2, ZNF469, HES6, FOXD1, GTF2I, NR5A1, and TP53, which are implicated in oxidative stress responses, astrocyte differentiation, and metabolic regulation, suggests that the observed secretome changes are under a sophisticated, coordinated transcriptional control. The ChEA3 analysis identified these top-ranking transcription factors based on their high combined scores, suggesting their strong predicted regulatory influence on the secretome gene set. This integration of metabolic and stress-related signaling cascades at the transcriptional level provides a deeper mechanistic understanding of astrocyte adaptation to iron deficiency.

We also observed changes in proteins involved in cell signaling, specifically CD9 antigen and RACK1, which may have implications for astrocyte-BBB communication. The decreased secretion of CD9, a tetraspanin involved in exosome release, hints at a potential modulation of astrocyte-BBB communication pathways. Exosomes are known mediators of intercellular signaling, and alterations in their protein cargo or release mechanisms could impact the expression of iron transport proteins on BMVECs. The downregulation of CD9 might indicate a shift in the specific signaling molecules being packaged into exosomes or a change in the overall rate of exosome secretion under low iron conditions, potentially altering the signals received by the BBB. The presence of RACK1, a known interactor with the iron mobilization-related protein NHE9 (Ohgaki et al., 2008), in the secretome, even with a modest (1.08-fold) and statistically insignificant fold change, also suggests a potential paracrine signaling mechanism. RACK1 could be involved in modulating NHE9 activity at the BBB, influencing iron mobilization from the blood. Further investigation into the exosomal content of astrocytes under varying iron levels and the precise role of RACK1 in astrocyte-BBB signaling is warranted.

While this study provides novel insights into astrocyte secretome alterations under iron-deficient conditions, several limitations should be acknowledged. First, the use of primary mouse astrocytes *in vitro*, while enabling controlled manipulation of iron levels, may not fully recapitulate the complex cellular interactions and systemic influences present *in vivo*, potentially affecting secretome profiles and BBB endothelial responses. Furthermore, the use of serum-free DMEM to induce iron deficiency, while necessary to control iron levels, introduces the potential for pleiotropic effects, including amino acid starvation, which could influence the observed secretome changes. Second, our experimental design collected the secretome exclusively during the 24-h period following the removal of the iron chelator, deferoxamine. This timing may have caused us to miss the initial secretome response that likely occurs during the early hours of active iron chelation when cellular stress is most acute. Consequently, our analysis was confined to a single time point, offering only a snapshot of the astrocytic response. While our approach effectively captures longer-term adaptations to iron deficiency, future studies should aim to collect secretome samples at earlier time points during deferoxamine treatment and across extended periods. This would provide a more comprehensive temporal understanding of astrocyte dynamics and their immediate and longer-term responses to iron chelation. Third, although proteomic analysis identified numerous secreted proteins, technical limitations inherent to LC-MS/MS may have prevented detection of all secretome components. Finally, the functional roles attributed to the identified proteins are based primarily on existing literature, and further experimental validation will be needed to confirm their contributions to astrocyte adaptation and iron regulation at the BBB.

In conclusion, this study provides the first comprehensive proteomic analysis of the astrocyte secretome under iron-deficient conditions, revealing a coordinated response involving proteins related to mitochondrial metabolism, iron transport, and cell signaling. Moving forward, it would be valuable to incorporate serum-supplemented conditions in future experiments to better isolate the specific effects of iron deficiency from those induced by serum withdrawal. The upregulation of Cp and downregulation of Ftl1 suggest that astrocytes actively promote iron availability during deficiency. Changes in signaling-related proteins such as CD9 and RACK1 further indicate potential paracrine communication with the BBB. Crucially, the detailed network and upstream regulator analyses highlight coordinated functional modules and predicted transcriptional control, significantly enhancing our understanding of the integrated astrocyte response. These findings advance understanding of astrocyte roles in brain iron homeostasis and identify potential therapeutic targets for disorders associated with iron imbalance. Future studies are needed to validate the functions of the identified proteins and to clarify the mechanisms by which astrocytes regulate iron transport across the BBB under physiological and pathological conditions.

## MATERIALS AND METHODS

### Cell culture and iron deficiency conditions

Primary astrocytes were isolated from neonatal C57BL/6 mouse pups (0–2 days old) by mechanical dissociation and enrichment using the shaking method, as previously described (Mosser et al., 2021). All animal procedures were approved by the Institutional Animal Care and Use Committee (IACUC) of the University of Michigan. Cells were cultured in DMEM high glucose medium (Gibco, catalog #11-965-084) supplemented with 10% heat-inactivated FBS (Gibco, catalog #A384010) and 1% penicillin/streptomycin (Gibco, catalog #15-140-148). For iron deficiency experiments, confluent astrocytes were washed with phosphate buffered saline (DPBS, Gibco, catalog #14040133) and incubated for 24 h in serum-free DMEM. Cells were then treated for 24 h with either serum-free DMEM (control) or serum-free DMEM containing 100 µmol/L deferoxamine (DFO; catalog #D9533, Sigma-Aldrich) (Simpson et al., 2015). After treatment, cells were rinsed and incubated with fresh serum-free DMEM for an additional 24 h to collect the conditioned medium.

### Sample preparation for mass spectrometry

Conditioned media proteins were concentrated using 3 kDa Corning Spin-X ultrafiltration columns and quantified by Qubit fluorometry (Invitrogen). The lowest yield, 18 µg (Control 2, sample IDs 52009 and 52982), was standardized across all six samples (three control, three iron-deficient) for downstream analysis.

Proteins were separated by one-dimensional SDS-PAGE on 5 cm, 10% Bis-Tris Novex mini-gels (Invitrogen) with MOPS buffer. Entire migration lanes were excised and divided into 20 bands, which were processed by automated in-gel digestion using the ProGest system (DigiLab), as previously described (Shevchenko et al., 2006). Bands were washed with 25 mM ammonium bicarbonate, dehydrated with acetonitrile, reduced with 10 mM DTT at 60°C for 1 h, alkylated with 50 mM iodoacetamide at room temperature for 1 h in the dark, and digested with sequencing-grade trypsin (Promega) at 37°C for 4 h. Digestions were quenched with formic acid, and peptide supernatants were collected for direct LC-MS/MS analysis without further desalting.

### Mass spectrometry

Half of each peptide digest was analyzed by nano LC-MS/MS using a Waters M-Class UPLC system coupled to a Thermo Fisher Scientific Fusion Lumos Tribrid mass spectrometer. Peptides were loaded onto a µ-Precolumn C18 PepMap100 trapping column and separated on a 75 µm×15 cm analytical column packed with 3 µm Luna C18 resin (Phenomenex) using a gradient of 0.1% formic acid in water (solvent A) and 0.1% formic acid in acetonitrile (solvent B) at 350 nl/min. Data were acquired in data-dependent acquisition (DDA) mode. Full MS scans were recorded at 60,000 FWHM resolution at m/z 400; the most intense precursor ions were fragmented by higher-energy collisional dissociation (HCD), with MS/MS scans acquired at 15,000 FWHM resolution. Dynamic exclusion was enabled to minimize repeated precursor selection. Instrument run time was approximately 10 h per sample.

### Data processing

Raw mass spectrometry data were processed using Mascot and searched against the SwissProt Mouse database supplemented with reversed sequences for false discovery rate (FDR) estimation and common contaminants (Perkins et al., 1999; Elias and Gygi, 2007; UniProt Consortium, 2025). Search parameters specified trypsin/P digestion (allowing up to two missed cleavages), with carbamidomethylation of cysteine as a fixed modification and oxidation (M), N-terminal acetylation, pyroglutamate formation (Q), and deamidation (N, Q) as variable modifications. Monoisotopic mass values were used, with peptide mass tolerance set at ±10 ppm and fragment mass tolerance at ±0.02 Da. Mascot DAT files were imported into Scaffold for peptide and protein validation (1% FDR at both levels) and to generate a non-redundant protein list, requiring at least two unique peptides per protein (Searle, 2010). Protein quantification was performed using the Normalized Spectral Abundance Factor (NSAF) method in Scaffold.

### Pathway enrichment analysis

Pathway enrichment analysis of differentially expressed proteins was performed using multiple tools (Szklarczyk et al., 2019; Kanehisa et al., 2017; Chen et al., 2013; Gene Ontology Consortium, 2021; D'Eustachio, 2011). In STRING (string-db.org), proteins were mapped to Mus musculus and analyzed for KEGG pathway enrichment, applying the default confidence threshold and considering FDR-adjusted *P*-values<0.05 (Benjamini-Hochberg method) as significant. Enrichr (maayanlab.cloud/Enrichr/) was used to identify enriched GO terms (Biological Process, Molecular Function, and Cellular Component) and Reactome pathways with FDR<0.05. g:Profiler (biit.cs.ut.ee/gprofiler/) analyzed GO and KEGG categories for annotated mouse genes using g:SCS correction and a significance cutoff of *P*<0.05, highlighting driver GO terms. WebGestalt (webgestalt.org) applied GSEA for KEGG pathway enrichment, mapping all 27 proteins to EntrezGene IDs using parameters of minimum category size 4, maximum 2000, and 1000 permutations.

These analyses enabled functional categorization of astrocyte secretome changes under iron deficiency, highlighting pathways related to iron metabolism, mitochondrial function, and oxidative stress.

### Secretion pathway prediction

To predict the potential secretion pathways of the identified differentially expressed proteins, the amino acid sequences for each protein were retrieved from the NCBI Protein database (https://www.ncbi.nlm.nih.gov/protein). These sequences were then analyzed using the SecretomeP 2.0 server (cbs.dtu.dk/services/SecretomeP/) (Bendtsen et al., 2004). Proteins with an NN score greater than 0.6 were considered strong candidates for non-classical secretion, consistent with the recommended threshold for high-confidence predictions.

### Network and upstream regulator analyses

To investigate the functional connectivity and upstream regulatory control of the differentially secreted proteins, two distinct network analyses and a transcription factor enrichment analysis were performed.

#### PPI network analysis (STRING and MCL clustering)

A PPI network of the 27 differentially secreted proteins was constructed using the STRING database (string-db.org, version 11.5). Proteins were mapped to *Mus musculus*, and interactions were retrieved based on a high confidence score (default setting, usually 0.700). The resulting network was then subjected to MCL within STRING to identify densely connected functional modules or clusters (Szklarczyk et al., 2019; Enright et al., 2002).

#### GeneMANIA network analysis

To further explore the broader functional associations and co-expression patterns among the 27 differentially secreted proteins, GeneMANIA (genemania.org) was utilized (Warde-Farley et al., 2010). The analysis integrated various data sources including co-expression, physical interactions, co-localization, pathway, and predicted functional associations. The network was generated with default settings, and the contribution of each interaction type was quantified.

#### Transcription factor enrichment analysis (ChEA3)

Upstream transcriptional regulators of the secretome gene set were predicted using the ChEA3 (ChIP-X Enrichment Analysis) platform (maayanlab.cloud/chea3/) (Keenan et al., 2019). The analysis was performed on the list of 27 differentially secreted proteins, leveraging multiple integrated libraries including ENCODE ChIP-seq, ReMap ChIP-seq, GTEx gene co-expression, ARCHS4 gene co-expression, and literature-curated TF-target interactions. Transcription factors were ranked based on their combined scores, which reflect the overall enrichment across these diverse datasets.

## Supplementary Material

10.1242/biolipen.062057_sup1 Supplementary information

Table S1.

Table S3.

Table S4.
