# Supplementary material for: Astrocyte secretome remodeling under iron deficiency: potential implications for brain iron homeostasis
Source: Biol Open. 2025 Jul 9;14(7):bio062057. doi: 10.1242/bio.062057 (PMC12309891; doi:10.1242/bio.062057)
Supplement: Supplementary information [file biolipen-14-062057-s1.pdf]

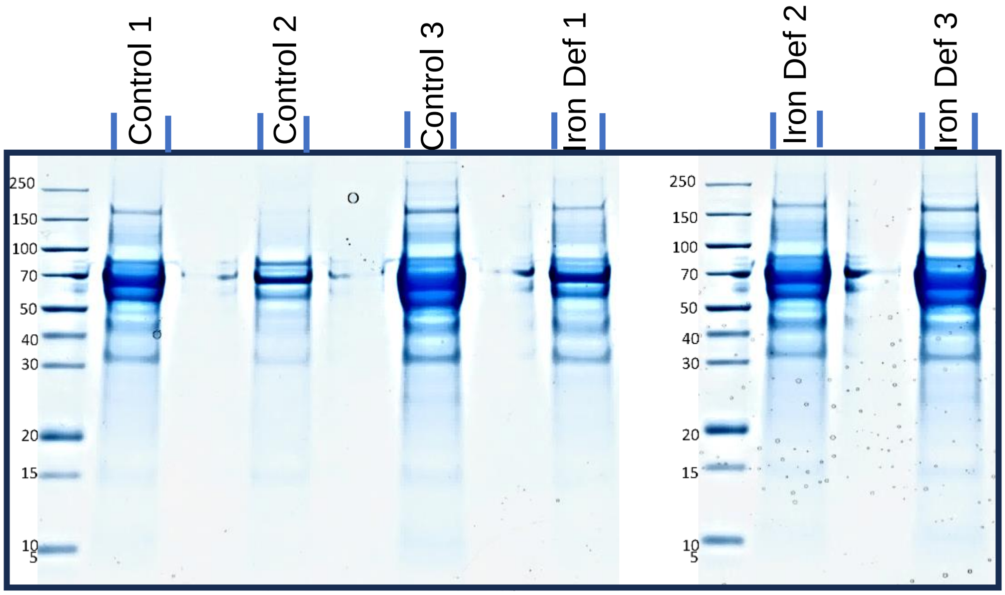

Fig. S1. SDS-PAGE analysis of conditioned media from primary mouse astrocytes. Conditioned media from astrocytes cultured in iron-deficient (Iron Def) and control conditions were collected in triplicates (Control 1-3, Iron Def 1-3). Each sample containing 18 µg of protein was resolved by SDS-PAGE on a 10% Bis-Tris Novex mini-gel (Invitrogen) using the MOPS buffer system. Molecular weight markers in kDa are indicated on the left side of each gel image.

Table S1.

Available for download at
https://journals.biologists.com/bio/article-lookup/doi/10.1242/bio.062057#supplementary-data

Biology Open • Supplementary information

Table S2. SecretomeP results.

Protein	NN-Score	Odds	Weighted by prior	Signal peptide prediction (by SignalP)
**Trafficking protein particle complex subunit 3**	0.528	1.238	0.002	
**Cytosolic NADP-isocitrate dehydrogenase**	0.563	1.234	0.002	
**Glucosamine-6-phosphate deaminase 1**	0.448	0.862	0.002	
**Major prion protein**	0.284	0.579	0.001	Predicted
**Nidogen-1**	0.676	1.881	0.004	Predicted
**Complement C1q subcomponent subunit B**	0.897	5.710	0.011	Predicted
**Argininosuccinate synthase**	0.374	0.700	0.001	
**Glyceraldehyde-3-phosphate dehydrogenase**	0.541	1.149	0.002	
**Ferritin light chain 1**	0.385	0.727	0.001	
**CD9 antigen**	0.439	1.349	0.003	Predicted
**Septin-2**	0.193	0.382	0.001	
**Small ribosomal subunit protein RACK1**	0.465	0.921	0.002	
**Transitional endoplasmic reticulum ATPase**	0.163	0.330	0.001	
**Ceruloplasmin**	0.632	1.563	0.003	Predicted
**Brevican core protein**	0.444	0.856	0.002	Predicted
**Neuroblastoma suppressor of tumorigenicity 1**	0.372	0.691	0.001	Predicted
**Tripeptidyl-peptidase 2**	0.369	0.692	0.001	
**Tropomyosin alpha-4 chain**	0.485	1.022	0.002	
**Inter-alpha-trypsin inhibitor heavy chain H5**	0.632	1.571	0.003	Predicted
**Importin-5**	0.627	1.648	0.003	
**Inactive tyrosine-protein kinase 7**	0.436	0.862	0.002	Predicted
**Meteorin**	0.925	6.464	0.013	Predicted
**Complement C1q tumor necrosis factor-related protein 5**	0.842	4.094	0.008	Predicted
**Splicing factor 3B subunit 3**	0.650	1.693	0.003	
**Protein slit-like 2**	0.179	0.359	0.001	Predicted
**Citrate synthase**	0.611	1.510	0.003	
**Inosine triphosphate pyrophosphatase**	0.483	0.969	0.002	

Non-classically secreted proteins should obtain an NN-score / SecP score exceeding the threshold, but not at the same time be predicted to contain a signal peptide. The recommended thresholds are **0.5** for bacterial sequences and **0.6** for mammalian sequences.

Biology Open • Supplementary information

Table S3.

Available for download at
https://journals.biologists.com/bio/article-lookup/doi/10.1242/bio.062057#supplementary-data

Table S4.

Available for download at
https://journals.biologists.com/bio/article-lookup/doi/10.1242/bio.062057#supplementary-data

Biology Open • Supplementary information
